# Herpes Simplex Virus 1 (HSV-1) ICP22 Protein Directly Interacts with Cyclin-Dependent Kinase (CDK)9 to Inhibit RNA Polymerase II Transcription Elongation

**DOI:** 10.1371/journal.pone.0107654

**Published:** 2014-09-18

**Authors:** Justyna Zaborowska, Sonja Baumli, Clelia Laitem, Dawn O'Reilly, Peter H. Thomas, Peter O'Hare, Shona Murphy

**Affiliations:** 1 Sir William Dunn School of Pathology, University of Oxford, Oxford, United Kingdom; 2 Northern Institute for Cancer Research, Newcastle University, Newcastle upon Tyne, United Kingdom; 3 Department of Oncology, Weatherall Institute of Molecular Medicine, University of Oxford, John Radcliffe Hospital, Oxford, United Kingdom; 4 Section of Virology, Faculty of Medicine, Imperial College, St Mary's Medical School, London, United Kingdom; University of Hyderabad, India

## Abstract

The Herpes Simplex Virus 1 (HSV-1)-encoded ICP22 protein plays an important role in viral infection and affects expression of host cell genes. ICP22 is known to reduce the global level of serine (Ser)2 phosphorylation of the Tyr1Ser2Pro3Thr4Ser5Pro6Ser7 heptapeptide repeats comprising the carboxy-terminal domain (CTD) of the large subunit of RNA polymerase (pol) II. Accordingly, ICP22 is thought to associate with and inhibit the activity of the positive-transcription elongation factor b (P-TEFb) pol II CTD Ser2 kinase. We show here that ICP22 causes loss of CTD Ser2 phosphorylation from pol II engaged in transcription of protein-coding genes following ectopic expression in HeLa cells and that recombinant ICP22 interacts with the CDK9 subunit of recombinant P-TEFb. ICP22 also interacts with pol II *in vitro*. Residues 193 to 256 of ICP22 are sufficient for interaction with CDK9 and inhibition of pol II CTD Ser2 phosphorylation but do not interact with pol II. These results indicate that discrete regions of ICP22 interact with either CDK9 or pol II and that ICP22 interacts directly with CDK9 to inhibit expression of host cell genes.

## Introduction

Herpesviruses are significant human pathogens associated with a range of mild to severe diseases. Although Herpes Simplex Virus (HSV) infections are generally self-limiting in healthy individuals, they can cause inflammation of the brain and eye and pose a significant mortality risk to infants and immuno-compromised adults [1]–[5].

The HSV ICP22 protein (encoded by the U_S_1 gene) is required for efficient viral replication, infection and latency in cell and animal models and regulates expression of some viral late genes [6]–[9]. ICP22 also counteracts the effect of IFN-β and has been implicated in the down-regulation of cellular genes [9]–[12]. Homologues of ICP22 are encoded by all alphaherpesviruses. An N-terminal-truncated protein called U_S_1.5 is also expressed from the U_S_1 gene [12]. Translation of U_S_1.5 has been variously reported to initiate at an ATG within residues 90–92, 171–173 or 147–149 [13]–[15] and functional differences between ICP22 and U_S_1.5 have not yet been completely characterized. However, U_S_1.5 is dispensable for both efficient acute virus replication and virus-induced chaperone-enriched (VICE) domain formation [9].

Recently, Kolb and colleagues identified a conserved region from residues 193 to 256 encompassing motifs predicted to be cyclin binding sites [2].

The carboxyl-terminal domain (CTD) of the large subunit of RNA polymerase II (pol II) comprises multiple repeats of the consensus sequence Tyr1Ser2Pro3Thr4Ser5Pro6Ser7 and all three serines (Ser) undergo dynamic phosphorylation and dephosphorylation during transcription [16]–[24]. Ectopic expression of ICP22 causes the loss of phosphorylation of Ser2 [25], a mark associated with productive elongation [19], [23]. Positive transcription elongation factor b (P-TEFb), which comprises the CDK9 kinase and a Cyclin T subunit, is thought to be a major CTD Ser2 kinase. There is evidence that ICP22 physically interacts with P-TEFb [26], [27] and it has been suggested that ICP22 inhibits CDK9 kinase activity [28]. However, it has not yet been definitively demonstrated whether ICP22 interacts with P-TEFb directly. In addition, the effect of ICP22 on expression of host cell genes has not been fully characterized.

Here we show that ICP22 expression causes loss of phosphorylation of Ser2 and Tyr1 of the pol II CTD heptapeptide repeat without markedly affecting phosphorylation of Ser5 and Ser7. These results are in line with the effect of ICP22 expression in other systems. Chromatin immunoprecipitation (ChIP) analysis indicates that pol II association with host cell genes and CTD Ser2 phosphorylation on transcriptionally-engaged pol II are drastically affected by ectopic expression of either full-length ICP22 or a truncated version comprising the conserved domain from amino acids 193 to 256. GST-mediated pull-down assays using recombinant ICP22 and recombinant P-TEFb indicate that these two proteins interact directly and that residues 193 to 256 of ICP22 are sufficient for this interaction. Moreover, a distinct region of ICP22 is required for interaction with pol II in HeLa nuclear extracts. On the basis of these results, we propose that ICP22 interacts with P-TEFb directly and with pol II, either directly or indirectly, to downregulate expression of host cell genes and facilitate the viral life cycle.

## Results

### Transient ectopic expression of ICP22 causes specific loss of pol II CTD Ser2 and Tyr1 phosphorylation

**Figure 1:**
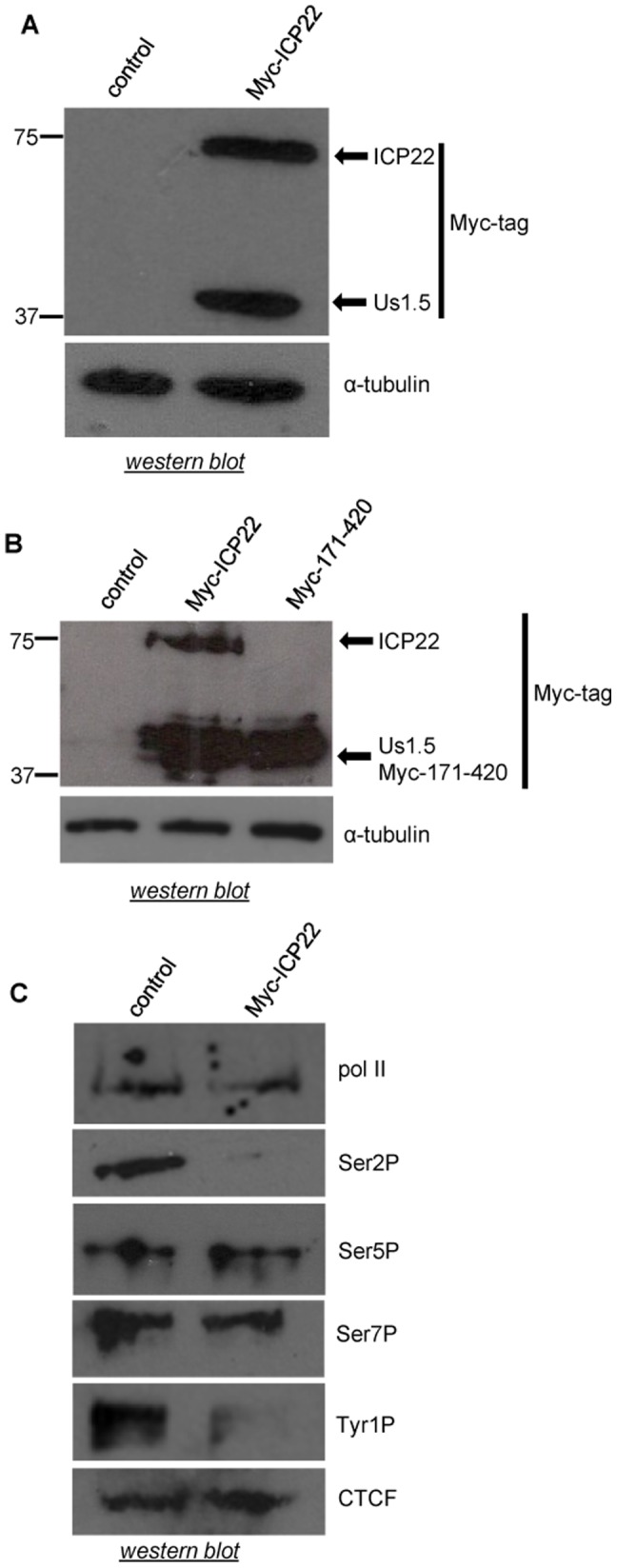
Transient ectopic expression of ICP22 causes specific loss of pol II CTD Ser2 and Tyr1 phosphorylation. (**A**) Myc epitope-tagged ICP22 and U_S_1.5 were ectopically expressed in HeLa cells from a transfected expression vector containing the ICP22 open-reading frame followed by three Myc epitope tags. pcDNA3 was used as the control in this and all subsequent transfections. An anti-Myc tag antibody was used for the western blot analysis and the positions of full-length ICP22 and U_S_1.5 are noted. α-tubulin was used as a loading control (**B**) Western blot analysis was performed as described in (A). The antibody used is indicated on the right. (**C**) Western blot analysis was carried out using antibodies to the CTD phosphorylation marks indicated on the right. CTCF was used as a loading control.

Full length ICP22 and the shorter form, Us1.5 are efficiently expressed from an expression vector containing the ICP22 open reading frame followed by three tandem Myc epitope tags after transient transfection into HeLa cells (Figure 1A). An N-terminal-truncated protein comprising residues 171 to 420 migrates the same as Us1.5, (Figure 1B) supporting the notion that translation of Us1.5 initiates at the AUG at residues 171-173 in HeLa cells, as noted in other cases [14]. In line with previous studies [25], ICP22 (and/or Us1.5) expression causes loss of phosphorylation of Ser2 of the pol II CTD, whereas phosphorylation of Ser5 is unaffected (Figure 1C). In addition, phosphorylation of the other serine in the CTD heptapeptide, Ser7, which also occurs during transcription [19], [29]–[31], is not affected (Figure 1C). However, phosphorylation of Tyr1 is reduced (Figure 1C).

These results indicate that ICP22 specifically affects the global levels of phosphorylation of the serine at position 2 and the tyrosine at position 1 of the pol II CTD heptapeptide in HeLa cells.

**Figure 2:**
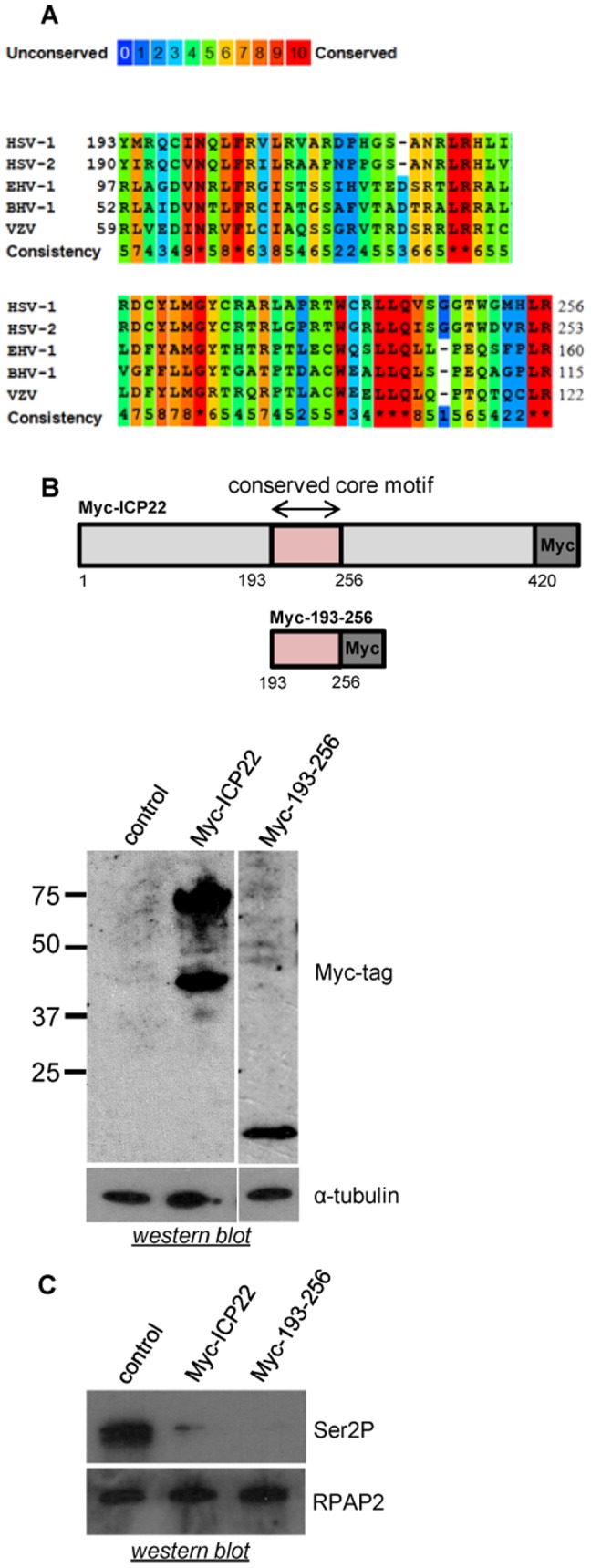
Ectopic expression of ICP22 or the 193–256 subdomain causes loss of pol II CTD Ser2 phosphorylation. Alignment of the conserved core motif within ICP22 of the alphaherpesviruses showing the degree of conservation according to the scheme above. Alignments were generated using PRALINE (http://www.ibi.vu.nl/programs/pralinewww/). Asterisks indicate identical residues. Accession Nos. AEDO2597, AEV91400, AAA46092, CAA54262, AEL30878 (**B**) Top, diagram of full-length ICP22 and 193–256 showing the position of the alphaherpesvirus conserved core motif and the epiptope tags (Myc). Bottom, the results of western blot analysis of extracts from HeLa cells transfected with the constructs indicated using antibodies to the Myc tag. α-tubulin was used as a loading control. (**C**) Western blot analysis of whole-cell extract from HeLa cells transfected with the constructs indicated using antibodies to the Ser2P CTD phosphorylation mark. RPAP2 was used as a loading control.

Amino acids 193 to 256 of ICP22 delimit a region conserved in the alpha-herpesviruses (Figure 2A), which contains predicted cyclin-binding domains [2]. Expression of this subdomain of ICP22 region causes loss of Ser2 phosphorylation of the pol II CTD as effectively as the full-length ICP22 (Figure 2B and 2C).

### Ectopic expression of ICP22 or the 193–256 subdomain causes loss of Ser2 phosphorylation from pol II transcribing host cell protein-coding genes

**Figure 3:**
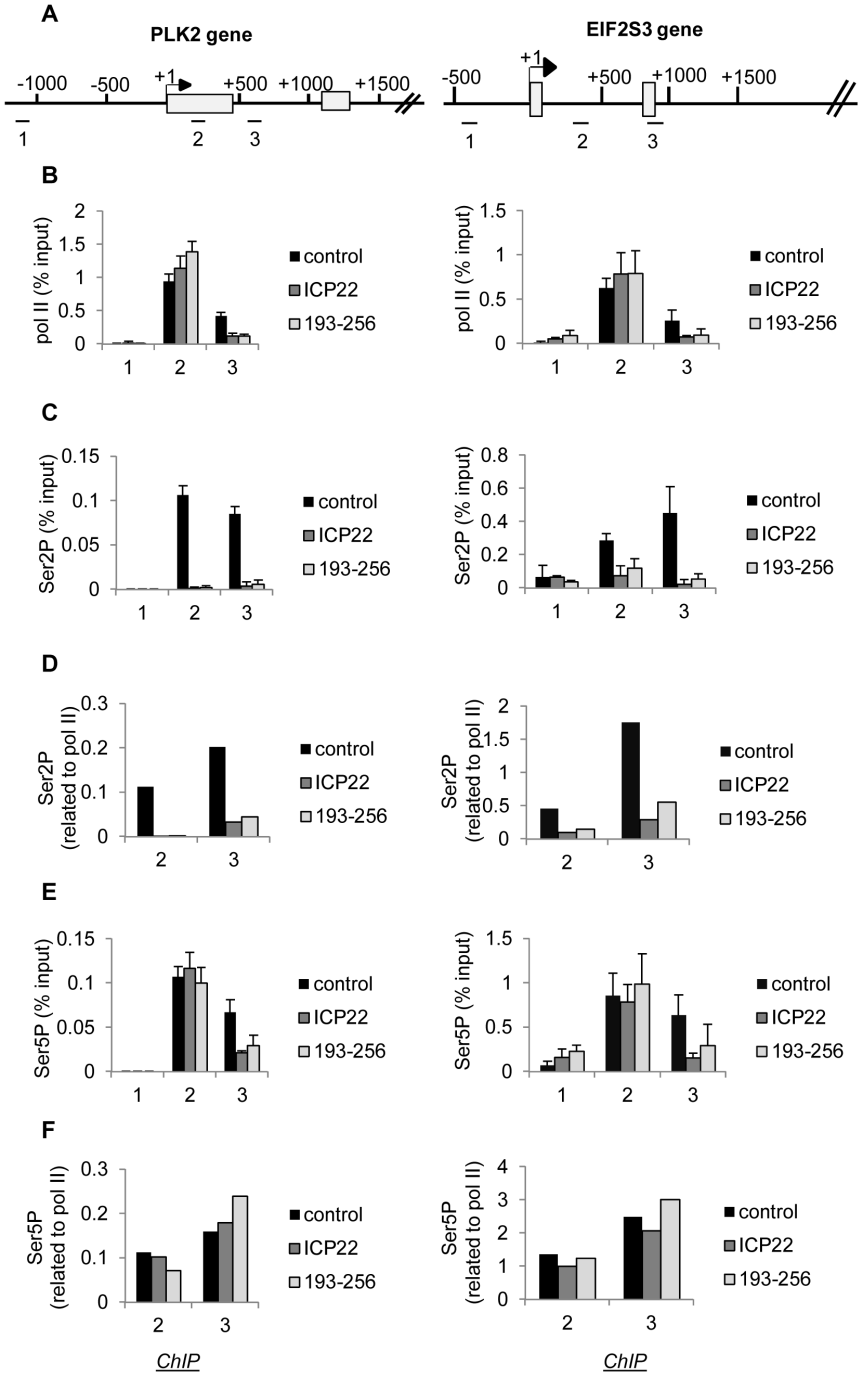
Ectopic expression of ICP22 or the 193–256 subdomain causes loss of Ser2 phosphorylation from pol II transcribing host cell protein-coding genes. (**A**) Diagrams of the PLK2 and EIF2S3 genes, with the position of chromatin immunoprecipitation (ChIP) primer pairs indicated. (**B**), (**C**), (**E**) The results of ChIP analysis using the antibodies indicated on the left after transfection of vectors expressing the Myc-tagged proteins indicated. (**D**), (**F**) The ratio of the CTD modification to pol II as indicated at the left.

ICP22 expression is associated with a decrease in the steady-state level of RNA from a number of host cell genes [32]. However, the effect of ICP22 expression on CTD phosphorylation of transcriptionally-engaged pol II has not yet been determined. We carried out chromatin immunoprecipitation (ChIP) analyses of pol II and pol II CTD Ser2 and Ser5 phosphorylation on two candidate genes, PLK2 and EIF2S3 (Figure 3A) after transfection of vector (pcDNA3), the whole ICP22 coding region or the 193–256 subdomain (Figure 3B–F). In untransfected cells and cells transfected with pcDNA3, pol II levels are high near the promoter (primer 2) and considerably lower further into the gene (primer 3) (Figure 3A and Figure S1 in File S1), which is the typical pattern of pol II association with human protein-coding genes [20], [33]. Pol II levels associated with the gene body are severely reduced on both genes following expression of either full-length Myc-ICP22 or Myc-193-256 (Figure 3B), suggesting that these proteins inhibit pol II-dependent transcription. As pol II levels close to the transcription start site are not adversely affected, transcription elongation rather than initiation of transcription appears to be affected. Ser2 phosphorylation of the CTD of transcriptionally-engaged pol II is also drastically reduced following expression of full-length Myc-ICP22 and Myc-193-256 (Figure 3C, D). In contrast, pol II CTD Ser5 phosphorylation is not adversely affected (Figure 3E, F).

The drastic loss of Ser2 phosphorylation from the CTD of pol II associated with the PLK2 gene suggests that the level of transfection of ICP22 is high and that Myc-ICP22 and Myc-193-256 are expressed in most cells. ICP22 is reported to localize in the nucleus in transfected and infected cells [34]–[36]. We have therefore analysed HeLa cells transfected with empty pcDNA3 and Myc-ICP22 by immunofluorescence (IF) using an antibody to an N-terminal region of ICP22 to investigate both the level of transfection and the nuclear localization of ICP22 (Figure S2 in File S1). The level of transfection with Myc-ICP22 is very high (greater than 90%) and ICP22 appears to be localized predominantly in the nucleus, consistent with previous findings [34]–[36] and as expected for a protein associated with the transcription apparatus.

The level of Ser2 phosphorylation of the pol II CTD associated with primer pair 2 of the EIF2S3 gene is less affected than the same region of the PLK2 gene (Figure 3C), suggesting some gene-specific variability in the ability of ICP22 to inhibit CTD Ser2 phosphorylation.

The effects on pol II and pol II CTD Ser2 phosphorylation are similar to those caused by small molecule inhibitors of CDK9, like DRB [20]. In support of this notion, ectopic expression of ICP22 inhibits pol II association with the β-actin gene beyond +1000 bp and pol II CTD Ser2 phosphorylation (Figure S3 in File S1), as we have previously shown for DRB using the same cells [20]. These results indicate that both full-length ICP22 and the shorter form comprising amino acids 193–256 effectively inhibit CTD phosphorylation of pol II transcribing host cell genes and effectively reduce the level of pol II associated with the body of the PLK2, EIF2S3 and β-actin genes. The drop in pol II ChIP levels downstream of the promoter is consistent with inhibition of transcription elongation. The inhibition of pol II CTD Ser2 phosphorylation suggests that the effect on pol II levels could be due to inhibition of the CDK9 kinase activity of P-TEFb causing a defect in transcription elongation.

ICP22 and 193–256 also cause a drop in the level of intronic RNA from the EIF2S3 gene, as measured by quantitative reverse transcription (qRT)PCR with primers specific for intron 2 (Figure S4 in File S1). This is likely to reflect the loss of nascent transcripts, consistent with an effect on transcription elongation.

### ICP22 is associated with cellular genes but does not affect recruitment of CDK9

**Figure 4:**
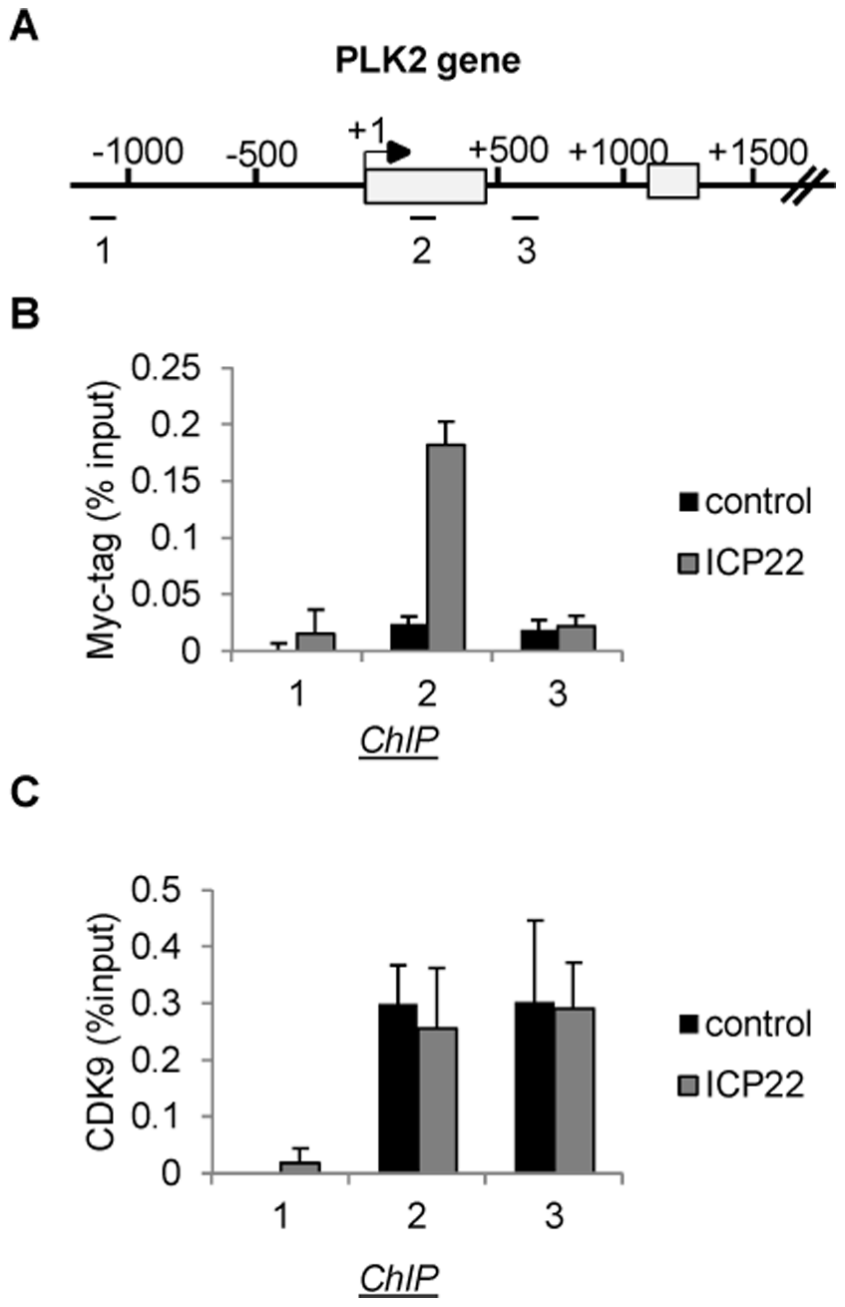
ICP22 is associated with cellular genes but does not affect recruitment of CDK9. (**A**) Diagram of the PLK2 gene, with the position of chromatin immunoprecipitation (ChIP) primer pairs indicated. (**B**), (**C**) ChIP analysis using the antibodies indicated on the left after transfection of vectors expressing the Myc-tagged ICP22 or pcDNA3 (control) as indicated.

Several different mechanisms could account for the effect of ICP22 on the level of pol II. For example, ICP22 could affect the recruitment of CDK9 to cellular genes. Alternatively, ICP22 could be recruited to genes to inhibit CDK9 activity at the site of transcription. To investigate these two possibilities, we carried out ChIP analysis on the PLK2 gene with an antibody to the Myc epitope-tag following expression of Myc-ICP22 (Figure 4). ICP22 is found associated with the PLK2 gene, close to the start site of transcription (Figure 4) in a region where pol II levels remain high after expression of ICP22 (Figure 3B and Figure S3B in File S1). ChIP analysis of CDK9 on the PLK2 gene following transient transfection of Myc-ICP22 indicates that ICP22 does not significantly alter the pattern of association of CDK9 (Figure 4C). The loss of pol II from the primer 3 region of the PLK2 gene caused by ICP22 (Figure 3A) does not cause a loss of CDK9, suggesting that CDK9 can be recruited to this gene independently of pol II. These results indicate that ICP22 is recruited to cellular genes and inhibits CDK9 at the site of transcription rather than blocking CDK9 recruitment.

### ICP22 interacts with pol II and CDK9

**Figure 5:**
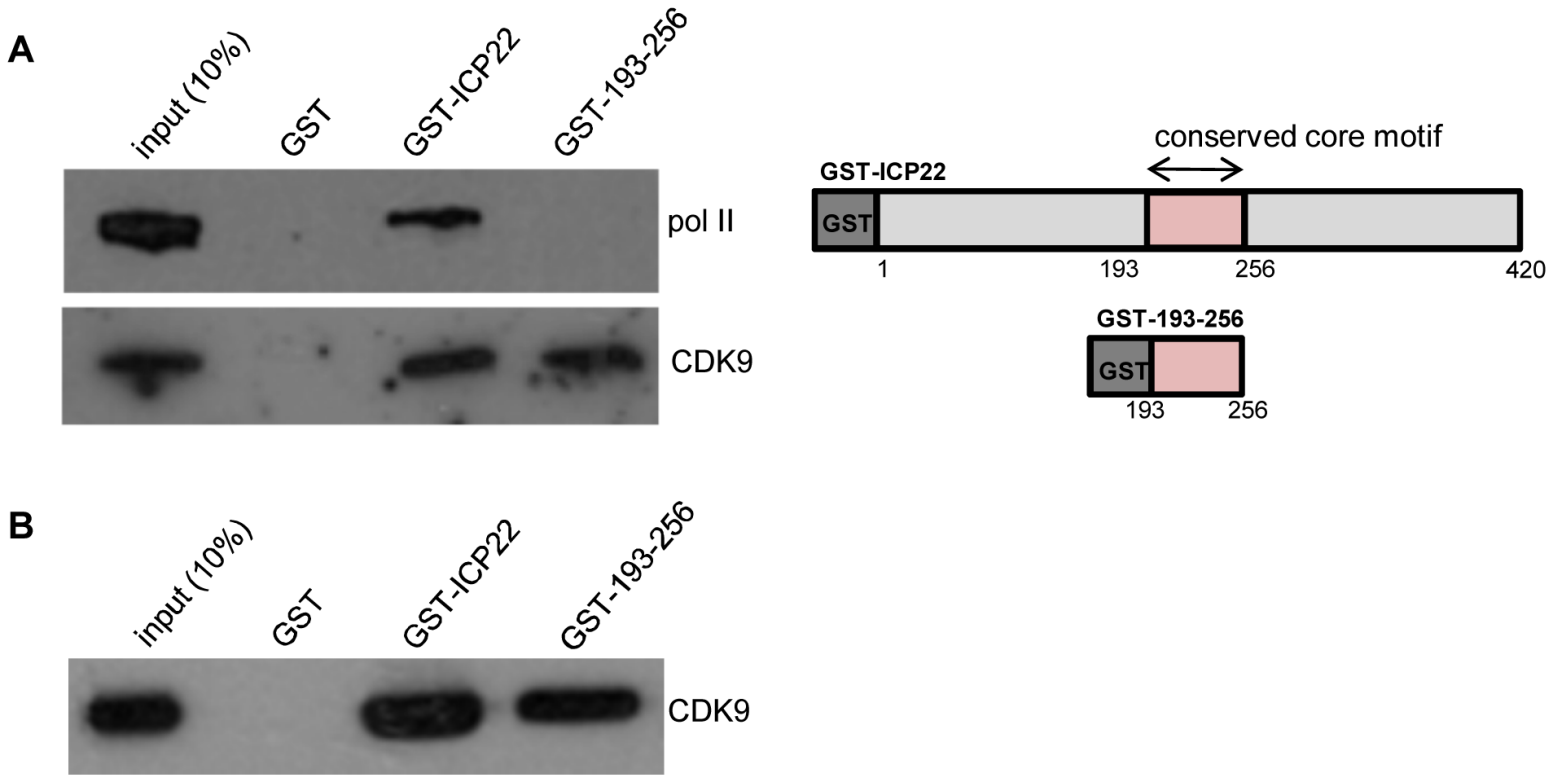
ICP22 interacts with pol II and CDK9. (**A**) GST-mediated pull-down from HeLa nuclear extract with the recombinant proteins indicated at the top, followed by western blot analysis using anti-pol II and anti-CDK9 antibodies. On the right, a diagram of full-length ICP22 and 193–256 shows the position of the alphaherpesvirus conserved core motif and the GST tags. (**B**) GST-mediated pull-down of recombinant P-TEFb by the recombinant proteins indicated at the top followed by western blot analysis using an anti-CDK9 antibody.

To further understand the molecular mechanism by which ICP22 affects pol II CTD Ser2 phosphorylation, we performed GST-ICP22- and GST-193-256-mediated pull-down, using either cell extract (Figure 5A) or recombinant P-TEFb (Figure 5B).

GST-ICP22 interacts with both CDK9 and pol II in cell extract whereas GST-193-256 interacts only with CDK9 (Figure 5A). Both recombinant full-length ICP22 and recombinant residues 193–256 interact with recombinant CDK9 (Figure 5B), indicating that the interaction between ICP22 and CDK9 is direct and that residues 193 to 256 are sufficient.

## Discussion

ICP22 is a multifunctional protein that plays important roles during HSV-1 infection [7], [8], [10] and homologues are likely to be important during the infection cycle of all alphaherpesviruses. Insights into the molecular mechanism of ICP22 function are critical for a full understanding of the role this protein plays in viral infection and the development of new anti-Herpes strategies that target ICP22. In addition, dissecting the interplay between ICP22 and the host transcriptional apparatus could shed light on key regulatory steps during transcription.

ICP22 was shown to cause loss of phosphorylation of Ser2 of the pol II CTD [37], a mark associated with productive transcription elongation [19]. P-TEFb is thought to be a major CTD Ser2 kinase [19] and it has been suggested that ICP22 interacts directly with P-TEFb to inhibit the activity of its CDK9 kinase [28]. We have confirmed that ICP22 causes specific loss of phosphorylation of Ser2 of the CTD without markedly affecting phosphorylation of Ser5 or Ser7 in HeLa cells. The specific loss of Ser2 phosphorylation therefore recapitulates the effect of ICP22 expression in other systems [25], [37]. These results also strengthen the conclusion that ICP22 does not affect the kinase activity of CDK7, which phosphorylates both Ser5 and Ser7 soon after initiation [23], [38], [39]. However, CTD Tyr1 phosphorylation is affected by ICP22. Tyr1 phosphorylation is thought to be carried out by c-Abl [30], [31] and the highest levels of Tyr1 phosphorylation are associated with promoters and enhancers [40], suggesting a role at an early stage of the transcription cycle. Inhibition of Tyr1 phosphorylation may play an important role in ICP22-mediated regulation of transcription of host and viral genes.

We show that the interaction between ICP22 and CDK9 is direct, suggesting a direct interaction with P-TEFb. Furthermore, our results indicate that the region of ICP22 conserved in alphaherpesviruses, residues 193–256, is sufficient for this interaction, suggesting that ICP22 from other alphaherpesviruses is also likely to interact with and inhibit P-TEFb. As the GST-ICP22 proteins we have used were purified from bacteria, they are unlikely to be post-translationally modified by, for example, phosphorylation, methylation, ubiquitylation in the same way as they would be when expressed in mammalian cells. Thus, no modification of ICP22 that would be added only during expression in mammalian cells is necessary for the interaction between ICP22 and CDK9. As the Cyclin T1 in the recombinant P-TEFb is truncated, our results further indicate that residues 1–266 of CyclinT1 are sufficient for any interaction with the P-TEFb complex. However, additional studies will be required to fully map region(s) of CDK9/Cyclin T1 involved in the interaction.

Our ChIP studies of the PLK2, EIF2S3 and β-actin genes indicate that CTD Ser2 phosphorylation on transcribing pol II is drastically affected by expression of either full-length ICP22 or the truncated form comprising residues 193–256. Our data suggest that transcription elongation rather than initiation of transcription is affected, consistent with the role of P-TEFb in overcoming an early-elongation checkpoint by phosphorylating the pol II CTD, NELF and DSIF [41]. Although the loss of pol II CTD Ser2 phosphorylation suggests that phosphorylation of NELF and DSIF is likely also to be inhibited, this remains to be determined.

Our finding that ICP22 inhibits CTD phosphorylation at the site of transcription without affecting CDK9 recruitment contrasts with the finding that ICP22 reduces Cyclin T1 association with the HSV-1 α, β and γ promoters in HSV-1 infected cells [27]. This may reflect differences in ICP22 action when it is ectopically expressed in cells on its own in our experiments and ICP22 action in the context of infection, differences in the effect on host cell and viral genes or that ICP22 has different effects on CDK9 and Cyclin T1 association.

Finally, our pull-down studies have also revealed a hitherto-unsuspected interaction between ICP22 and pol II that is likely to be important for the *in vivo* function of ICP22. As residues 193–256 do not interact with pol II, but inhibit pol II CTD Ser2 phosphorylation and pol II levels, the interaction between ICP22 and pol II does not appear to be required for CDK9 inhibition.

**Figure 6:**
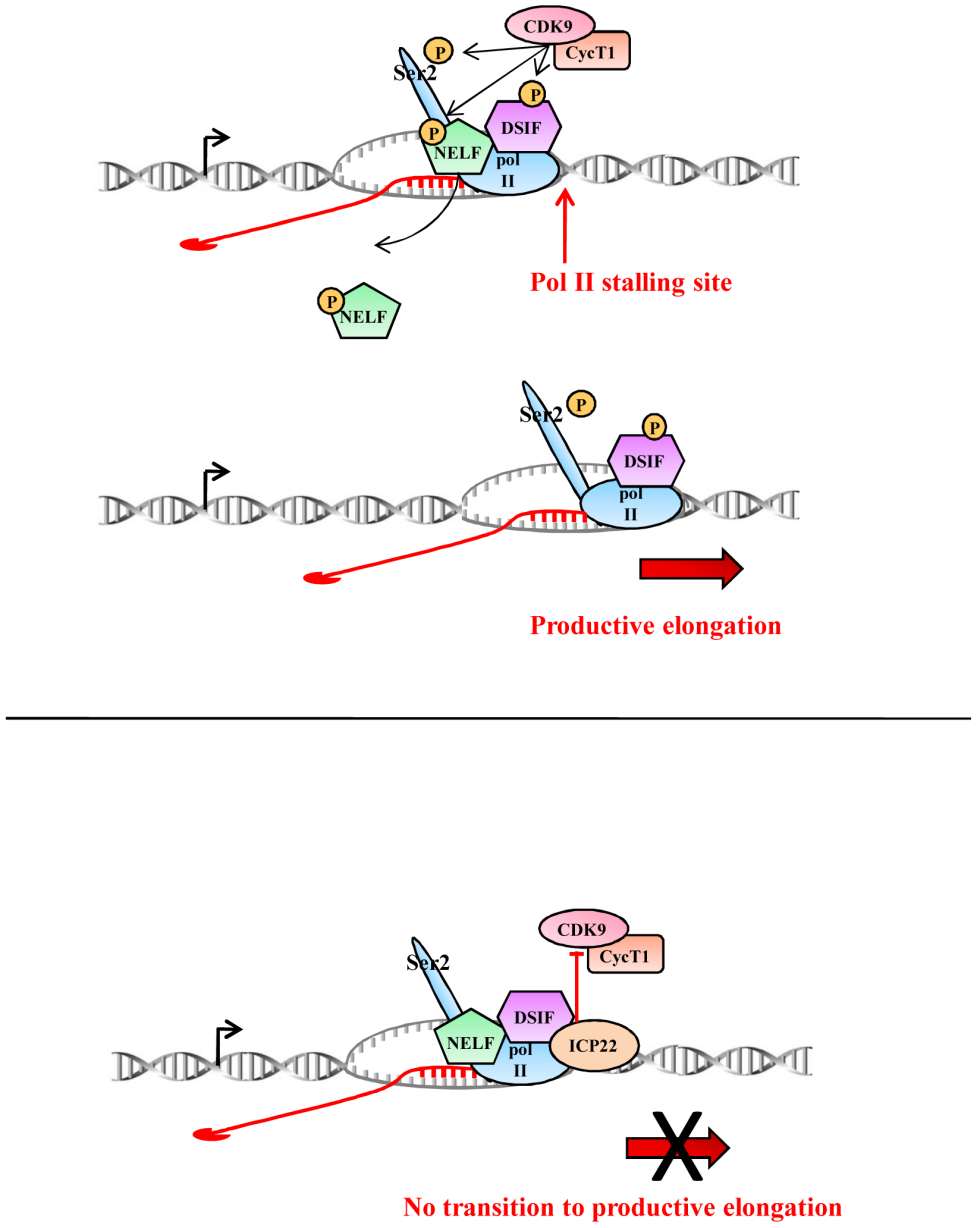
Model for the role of ICP22 in inhibition of pol II CTD Ser2 phosphorylation. In uninfected cells (top panel), the negative elongation factor (NELF) and the DRB-sensitivity-inducing factor (DSIF) enhance pol II stalling. Subsequent recruitment of P-TEFb allows phosphorylation of DSIF, NELF and Ser2 of the pol II CTD, which leads to productive elongation. In the context of HSV-1-infected cells (bottom panel), ICP22 associates with P-TEFb and inhibits the kinase activity of CDK9 at the site of transcription, as indicated by the loss of phosphorylation of Ser2 of the pol II CTD, NELF and DSIF. As a consequence, the transition to productive elongation is inhibited. Interaction between ICP22 and pol II is not necessary to recruit ICP22 to genes or inhibit CDK9 when ICP22 is ectopically expressed in cells on its own. However, the interaction between pol II and ICP22 may be necessary to recruit ICP22 to host cell genes in HSV1-infected cells. Alternatively, interaction between ICP22 and pol II may play a role in regulation of viral gene expression by ICP22.

On the basis of the data presented here, our current working model is that ICP22 is recruited to host cell genes where it directly associates with P-TEFb to inhibit the CDK9 kinase activity, which results in downregulation of expression of host cell genes at the level of transcription elongation (Figure 6). The level of pol II at the beginning of the PLK2 and EIF2S3 genes remains high after ICP22 expression, suggesting that pol II is poised here after CDK9 inhibition rather than released. Interaction between pol II and ICP22 could help recruitment of the full-length ICP22 to host cell genes when ICP22 levels are low, for example at the early stages of infection, or in the context of viral infection. Alternatively, the interaction between pol II and ICP22 may play a role in regulation of expression of viral genes. ICP22 has been shown to repress activation of reporter gene transcription by the HSV-1 ICP0 protein, to repress transcription from the HSV-1α, β and γ promoters and to upregulate expression of some HSV-1 late genes [9], [27], [42]. VP16 can overcome ICP22-mediated repression of the HSV-1 α promoter [27], emphasizing that ICP22 function can be modulated by the activity of other viral proteins. The interaction between ICP22 and pol II could therefore be important for either repression or activation of viral genes at specific stages of the viral life cycle.

## Materials and Methods

### Cell lines

HeLa cells were grown in DMEM medium supplemented with 10% fetal calf serum, 100 U/mL penicillin, and 2 mM L-glutamine at 37°C and 5% CO2.

### Expression Constructs

To generate Myc-ICP22 and Myc-193-256, three tandem Myc epitopes were cloned at the C terminus of the ICP22 coding region and residues 193–256 of ICP22 in pcDNA3. To make GST-ICP22 and GST-193-256, the coding regions were cloned into pGEX-6P-1. The identity of all plasmid constructs was verified by sequence analysis.

### RNA preparation

RNA was extracted from 6×10^6^ cells transfected with pcDNA3, Myc-ICP22 or Myc-193-256 HeLa cells using TRIzol (Invitrogen) according to the manufacturer's instructions. Reverse-transcription (RT) was performed with 1 µg of RNA using random hexamers with the SuperScriptIII kit (Invitrogen) according to the manufacturer's instructions. cDNA was amplified by qRT-PCR using QuantiTect SYBR Green PCR (Qiagen) using a Rotor-Gene RG-3000 (Corbett Research). The sequences of primers are given in Table 1.

**Table 1:** Sequence of primer sets used for qRT-PCR analysis.

Name	Sequence of forward primer	Sequence of reverse primer
PLK2 1	AAGTGTCTCCTCTGTACCAGGA	GGAATCATGACCAGGAAATGTACGG
PLK2 2	ACCGGGGTGTTGGGTGCTAGT	ATAGTCCGCAAAAGCTCCATG
EIF2S3 1	AACCAGCGAACTTCAGACGCT	GTCCCCAGCTTGTTCCCAGAGA
EIF2S3 2	GAGAAGCTGGAGTGACTCTAGG	CACTGACTAGTCCCAATACC
EIF2S3 3	GATGGTGGCAAGATGTAGATAGCA	CGTCAACTTGGTAACATCCTGCAATG
EIF2S3 4	TACAGGCCTTGAACTACTGC	CTTAGCATAGGTTGTTCGGAGG
5.8S	CAAGCGACGCTCAGACAGG	GTGGATCACTCGGCTCGTGC
β-actin 1	GCTGCGGCTGGGTAGGTTTG	CACTTAGAAGTCGCAGGACC
β-actin 2	GGGCAACCGGCGGGGTCTTT	ACGCAGTTAGCGCCCAAAGG
β-actin 3	CCCCATCGAGCACGGCATCGTC	CACCTGGGTCATCTTCTCGCGGT

### Chromatin Immunoprecipitation

HeLa cells were transfected with pcDNA3, Myc-ICP22 and Myc-193-256 vectors using Lipofectamine 2000 (Invitrogen) according to the manufacturer's instructions before being subjected to ChIP analysis as detailed [43]. ChIP samples were analyzed by qRT-PCR using QuantiTect SYBR Green PCR (Qiagen) and Rotor-Gene RG-3000 (Corbett Research). Error bars indicate the standard deviation from at least three independent experiments. Final ChIP values are expressed as a percentage of the total DNA input after deduction of the signal obtained using rabbit IgG as a negative control. Antibodies against pol II (sc-899) and CDK9 (sc-484) were obtained from Santa Cruz Biotechnology. Antibodies against Ser2P (ab5095), Ser5P (ab5131) and the Myc-tag (ab9132) were obtained from Abcam. The sequences of primers are given in Table 1.

### Western Blot Analysis

Western blotting was carried out as described previously [16] using proteins harvested from cells boiled in Laemmli buffer (50 mM Tris (pH6.8), 2% sodium dodecyl sulphate, 5% β-mercaptoethanol, 10% glycerol, 0.1% bromophenol blue) and antibodies against pol II diluted 1∶200 (sc-899, Santa Cruz), pol II CTD Ser2P diluted 1∶1000 (ab5095, Abcam), Ser5P diluted 1∶1000 (ab5131, Abcam), Ser7P diluted 1∶200 [29], Tyr1P diluted 1∶200 (61383, Active motif), RPAP2 diluted 1∶1000 (17401-1-AP, Proteintech), CTCF diluted 1∶1000 (07–729, Millipore), Myc-tag diluted 1∶1000 (ab9132, Abcam), CDK9 diluted 1∶500 (sc-484, Santa Cruz) and α-tubulin diluted 1∶1000 (200-301-880, Tebu-bio).

### Immunofluorescence

Cells for immunofluorescence were plated into 6-well trays containing one coverslip per well and transfected with pcDNA3 or Myc-ICP22 plasmid DNA using Lipofectamine 2000 according to the manufacture's instructions. Cells were washed with PBS and fixed for 15 min at room temperature with 4% PFA/2% sucrose in 1xPBS. The samples were then blocked and incubated for 1 h with the primary antibody diluted 1∶100 (Hhv11 ICP22 antibody, orb159247). Following 3× washes with PBS +0,1% Triton, the secondary antibody (Alexa Fluor 488 anti-rabbit, A21206) was added to the blocking solution and incubated for 30 minutes. Three additional washes were carried out before the coverslips were mounted (EverBrite Mounting with DAPI, Biotium 23002). Samples were then examined with a Zeiss AxioObserver Z1 20× and images processed using Fiji Image J [44] and images processed using Adobe Photoshop software. Dapi was detected with Zeiss Filter set 02 and Alexa Fluor 488 with Zeiss Filter set 38.

### Protein Purification and GST Pull-down Assays

GST-ICP22, GST-193-256 and GST were purified from Escherichia coli (Rosetta cells) and immobilized on Sepharose 4B-gluthatione beads (GE Healthcare). Hela nuclear extract (NE) was produced as described [45]. 400 µl of HeLa NE was incubated at 4°C for 2 h with immobilized GST-tagged protein or GST. The beads were washed with 1×0.15 M HEGN, 4×0.3 M HEGN, 1×0.15 M HEGN (HEGN-X buffer: XM KCl, 20 mM Hepes pH 7.6, 10% glycerol, 0.2% NP-40, 0.1 mM EDTA, 1 mM DTT, 1× complete inhibitors). Protein-bound beads were boiled and loaded directly on SDS-PAGE gels.

For the GST-mediated pull-down of recombinant P-TEFb, the recombinant P-TEFb was produced as described [46]. The GST-ICP22, GST-193-256 and GST proteins immobilized on beads were incubated with 300 ng of recombinant P-TEFb in 200 µl of buffer (250 mM NaCl, 50 mM Tris pH7.5, 1% NP40, 2 mM EDTA) at 4°C for 1 h. Beads were subsequently washed 3× and eluted with 10 mM glutathione. Eluted samples were analysed by western blot.

## Supporting Information

File S1 **Contains figures S1–S4.** Figure S1. Pol II levels are higher at the promoter than within the body of the PLK2 and EIF2S3 genes. Figure S2. Transient transfection of Myc- ICP22 in HeLa cells with Lipofectamine 2000 is efficient. Figure S3. ICP22 recapitulates the effect of DRB on pol II transcribing the β-actin gene. Figure S4. Ectopic expression of ICP22 or the 193–256 subdomain causes loss of intronic RNA.
(PDF)
