# Supplementary material for: Herpes Simplex Virus 1 (HSV-1) ICP22 Protein Directly Interacts with Cyclin-Dependent Kinase (CDK)9 to Inhibit RNA Polymerase II Transcription Elongation
Source: PLoS One. 2014 Sep 18;9(9):e107654. doi: 10.1371/journal.pone.0107654 (PMC4169428; doi:10.1371/journal.pone.0107654)
Supplement: File S1 — Contains figures S1–S4. Figure S1. Pol II levels are higher at the promoter than within the body of the PLK2 and EIF2S3 genes. Figure S2. Transient transfection of Myc- ICP22 in HeLa cells with Lipofectamine 2000 is efficient. Figure S3. ICP22 recapitulates the effect of DRB on pol II transcribing the β-actin gene. Figure S4. Ectopic expression of ICP22 or the 193–256 subdomain causes loss of intronic RNA. (PDF) [file pone.0107654.s001.pdf]

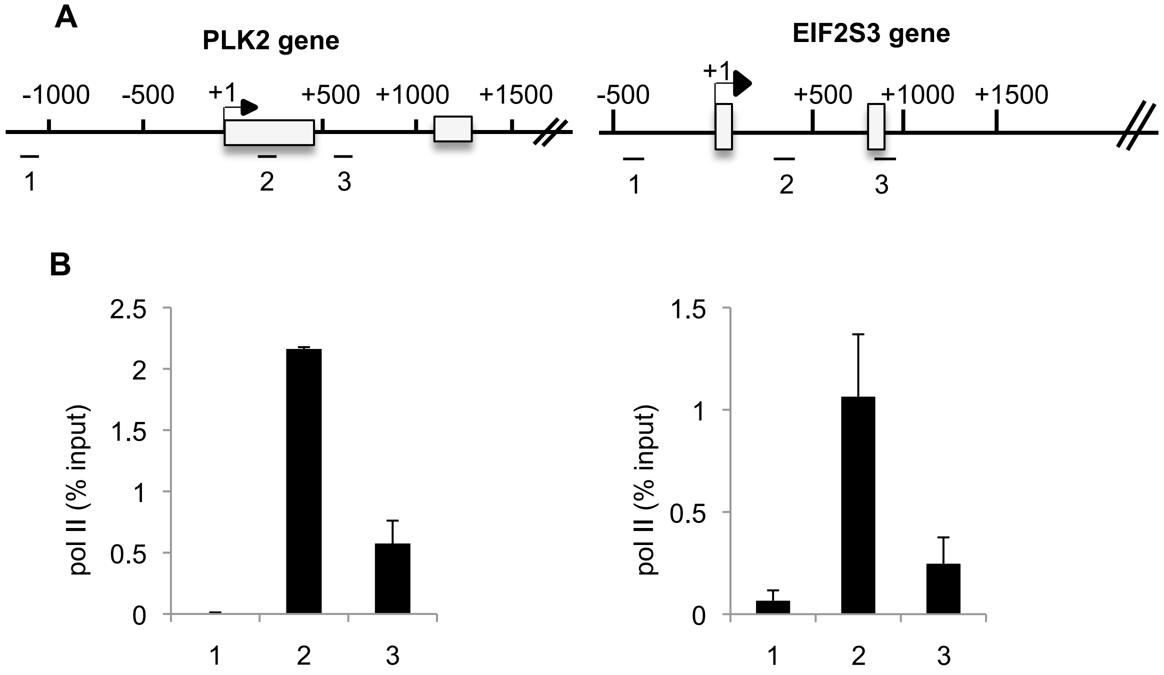

Figure S1. Pol II levels are higher at the promoter than within the body of the PLK2 and EIF2S3 genes. (A) Diagrams of the PLK2 and EIF2S3 genes, with the position of chromatin immunoprecipitation (ChIP) primer pairs indicated. **(B)** The results of ChIP analysis using an anti-pol II antibody as indicated.

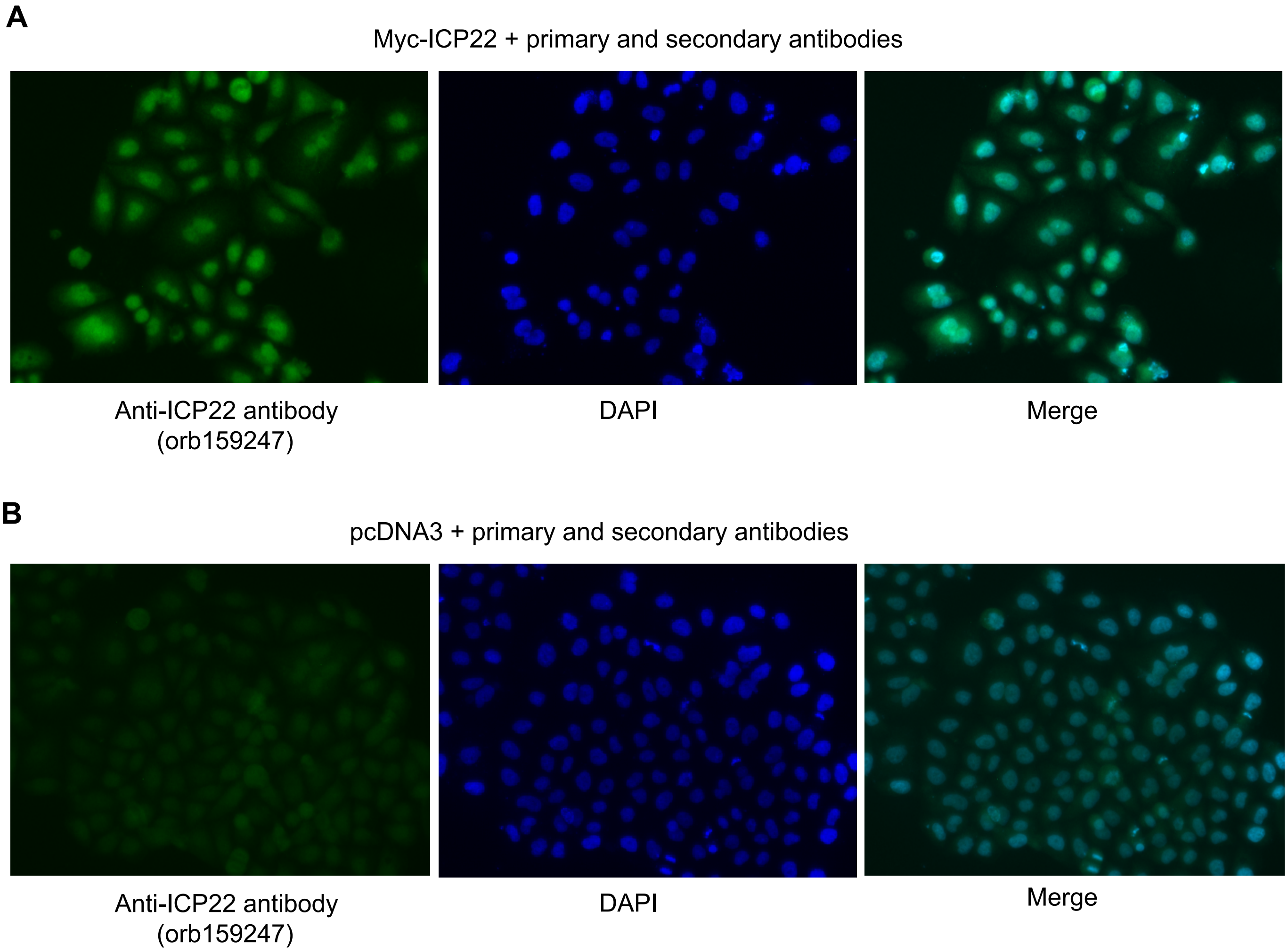

Figure S2. Transient transfection of Myc-ICP22 in HeLa cells with Lipofectamine 2000 is efficient. (A), (B) Expression of Myc-ICP22 in transfected HeLa cells. HeLa cells were transfected with the indicated plasmids and processed for immunofluorescence 15h post-transfection using an anti-ICP22 antibody. Representative cells are shown. The transfection efficiency with Lipofectamine 2000 was greater than 90%. The left panel shows fluorescence from the anti-ICP22 antibody plus the secondary (green), the middle panel shows DAPI staining of DNA (blue) and the right panel shows a merge of the left and right images. The plasmid transfected is noted above each set of panels.

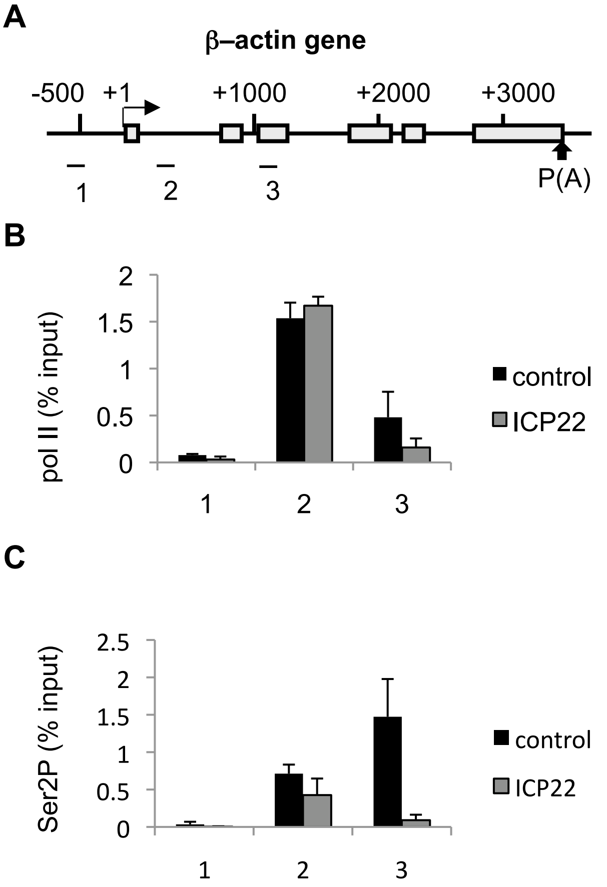

Figure S3. ICP22 recapitulates the effect of DRB on pol II transcribing the β-actin gene. (A) Diagram of the β-actin gene, with the position of chromatin immunoprecipitation (ChIP) primer pairs indicated. P(A) indicates the position of the polyadenylation site. **(B)**, **(C)** ChIP analysis using the antibodies indicated on the left after transfection of the vectors expressing the Myc-tagged ICP22 or pcDNA3 (control) as indicated.

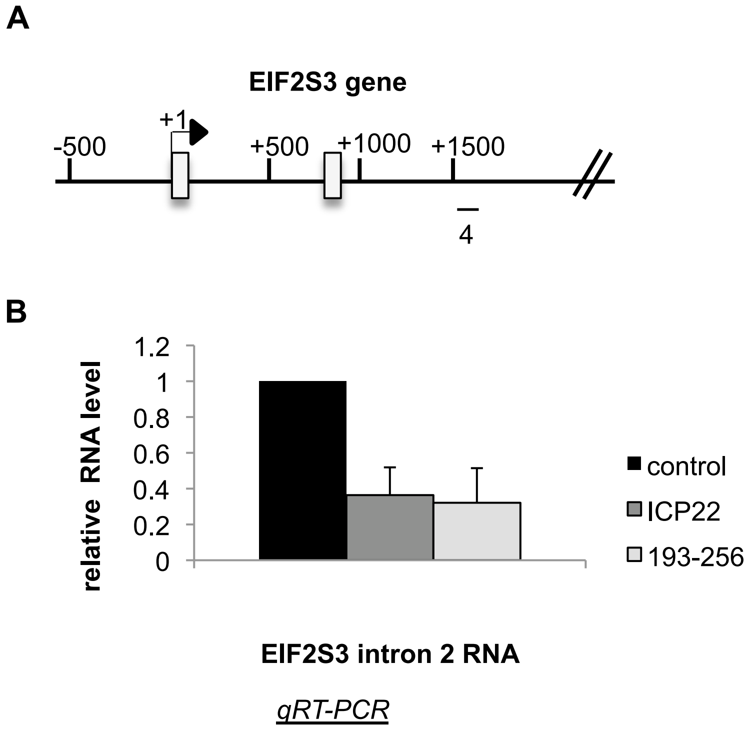

Figure S4. Ectopic expression of ICP22 or the 193-256 subdomain causes loss of intronic RNA (**A**) Diagram of the EIF2S3 gene, with the position of primers used for analysis indicated. **(B)** qRT-PCR analysis of intronic RNA from EIF2S3 after transfection of the vectors expressing the Myc-tagged ICP22, the Myc-tagged 193-256 subdomain or pcDNA3 (control) as indicated. RNA levels were normalised to the level of 5.8S RNA and the level for pcDNA3 transfected cells (control) taken as 1.
